# The Quality of Wheat Bread With Ultrasonicated and Fermented By-Products From Plant Drinks Production

**DOI:** 10.3389/fmicb.2021.652548

**Published:** 2021-03-17

**Authors:** Elena Bartkiene, Vadims Bartkevics, Iveta Pugajeva, Anastasija Borisova, Egle Zokaityte, Vita Lele, Vytaute Starkute, Paulina Zavistanaviciute, Dovile Klupsaite, Daiva Zadeike, Grazina Juodeikiene

**Affiliations:** ^1^Department of Food Safety and Quality, Faculty of Veterinary, Lithuanian University of Health Sciences, Kaunas, Lithuania; ^2^Faculty of Animal Sciences, Institute of Animal Rearing Technologies, Lithuanian University of Health Sciences, Kaunas, Lithuania; ^3^Centre of Food Chemistry, University of Latvia, Riga, Latvia; ^4^Institute of Food Safety, Animal Health and Environment “BIOR,” Riga, Latvia; ^5^Department of Food Science and Technology, Kaunas University of Technology, Kaunas, Lithuania

**Keywords:** wheat bread, plant drinks by-products, fermentation, ultrasonication, acrylamide, bread quality

## Abstract

During plant-based drinks production a significant amount of valuable by-products (BPs) is obtained. The valorization of BPs is beneficial for both the environment and the food industry. The direct incorporation of the fermented and/or ultrasonicated almond, coconut, and oat drinks production BPs in other food products, such as wheat bread (WB) could lead to the better nutritional value as well as quality of WB. Therefore, in this study, various quantities (5, 10, 15, and 20%) of differently treated [ultrasonicated (37 kHz) or fermented with *Lacticaseibacillus casei* LUHS210] almond, coconut, and oat drinks preparation BPs were used in wheat bread (WB) formulations. Microbiological and other quality parameters (acidity, color, specific volume, porosity, moisture content, overall acceptability) as well as bread texture hardness during the storage and acrylamide content in the WB were evaluated. Among the fermented samples, 12-h-fermented almond and oat, as well as 24-h-fermented coconut drinks preparation BPs (pH values of 2.94, 2.41, and 4.50, respectively; total enterobacteria and mold/yeast were not found) were selected for WB production. In most cases, the dough and bread quality parameters were significantly (*p* ≤ 0.05) influenced by the BPs used, the treatment of the BPs, and the quantity of the BPs. The highest overall acceptability of the WB prepared with 20% fermented almond drink preparation by-product (AP), 15% fermented oat drink preparation by-product (OP), and 15% ultrasonicated OP was established. After 96 h of storage, the lowest hardness (on average, 1.2 mJ) of the breads prepared with 5% fermented AP, coconut drink preparation by-product (CP), and OP and ultrasonicated CP was found. The lowest content of acrylamide in the WB prepared with OP was found (on average, 14.7 μg/kg). Finally, 15% fermented OP could be safely used for WB preparation because the prepared bread showed high overall acceptability, as well as low acrylamide content.

## Introduction

Food production by-products (BPs) may cause detrimental effects in the environment if no treatment, minimization, or prevention methods are undertaken. Nowadays, food processing BPs are considered a source of valuable compounds that could be recovered to fortify different products (Galanakis, 2020). The non-dairy plant-based drink industry is increasing (Munekata et al., 2020). However, during the technological production process, raw material (cereals, nuts, fruits) is extracted with water, and the solid part, usually called press cakes or meal, is generated in significant amounts. The formed BPs are still rich in dietary fibers, proteins, and macro- and micronutrients (Houmy et al., 2020; Sobczak et al., 2020). Moreover, the other compounds, such as phenols, flavonoids, or fatty acids, in the coconut press cake possess antidiabetic and anticancer properties, while oats are characterized by a high content of β-glucan and positively affect human health (Karandeep et al., 2019; Krochmal-Marczak et al., 2020). However, plant-based drinks and BPs could contain some antinutritional compounds. The *myo*-inositol phosphates in almonds and oats were found (Silva et al., 2020). Oxalate and phytates, which reduce the bioavailability of minerals and proteins, are found in oat while almonds contain allergenic protein amandin (Munekata et al., 2020). However, the antinutritional compounds in coconut were not found (Karandeep et al., 2019). Considering all that, the valorization of plant-based drink preparation BPs is beneficial for both the environment and the food industry.

However, for further use in the food industry, BPs must meet biosafety and chemical safety requirements, and different techniques could be used to achieve these. Fermentation with lactic acid bacteria (LAB) could be one of these methods because it not only enhances the organoleptic properties and nutritional value of fermented substrates, but it also improves their microbiological stability and possesses a detoxifying effect (Admassie, 2018). In addition, LAB are known as probiotics, which have beneficial effects on human health. Another technique called the ultrasound technique, which involves mechanical sound waves, could have mechanical and/or chemical effects on the various processes in food and could inactive microorganisms or enzymes (Gallo et al., 2018). The mentioned techniques are safe, sustainable and eco-friendly. Besides, our previous study showed that the bio-valorization (fermentation with the *Lacticaseibacillus casei* LUHS210 strain) and ultrasound treatment of almond, coconut, and oat drink preparation BPs could be useful for the BPs biosafety improving without negative changes in the chemical composition (Bartkiene et al., 2020a). Moreover, the antimicrobial activity of *L. casei* LUHS210 was confirmed by our previous research (Bartkiene et al., 2019).

The most effective valorization of by-products can be done without expensive intermediate treatment steps (extraction, drying, etc.), and we hypothesize that such direct incorporation of the fermented and/or ultrasonicated almond, coconut, and oat drink preparation BPs in other food products, such as wheat bread, is possible. Bread continues to be a significant part of daily human nutrition. Wheat bread has a high glycaemic index and contains gluten. Due to this, frequently consumed wheat bread may elicit some health-related issues such as insulin resistance or celiac disease (Kourkouta et al., 2017). To minimize these negative aspects, the nutritional value of wheat bread should be increased through changes in bread formulations. However, the addition of new ingredients to the main product recipe is challenging and can lead to non-desirable changes in the structure of bread, as well as acrylamide formation during the thermal processes, deteriorated sensory properties, etc. Most studies focus on the nutritional value of wheat bread increasing by including compounds that are more valuable than wheat flour to the main recipe. The acrylamide concentration in traditional wheat bread is not high; however, our previous studies showed that new ingredients addition to the main formula could lead to acrylamide formation (Bartkiene et al., 2013a, 2015, 2016). The tolerable daily intake (TDI) of acrylamide to avoid neurotoxicity is estimated at 40 μg/kg bw/day, and to avoid carcinogenic effects, 2.6 μg/kg bw/day is recommended (Tardiff et al., 2010). Elias et al. (2017) reported that the acrylamide concentration in cereal products is, on average 1390 μg/kg. The main factors affecting acrylamide formation in products are temperature and duration of food thermal treatment (Mencin et al., 2020). However, the formulation of a recipe with the lowest acrylamide precursors concentration and the selection of appropriate technological solutions to reduce concentration of these precursors are very important (Bartkiene et al., 2013a, 2015, 2016). Among the technologies used to reduce the acrylamide concentration in foods, reports have included lower processing temperatures, the removal of potential substrates (i.e., amino acids, reducing sugars), and the application of antioxidants (Constantinou and Koutsidis, 2016; Nasiri Esfahani et al., 2017). Also, different fermentation schemes to reduce acrylamide formation during bread preparation have been studied (Bartkiene et al., 2017). Finally, by including new ingredients in the main bread formula, in addition to the main bread quality parameters, acrylamide formation must be monitored.

In this study, the challenges associated with the application of almond, coconut, and oat drinks preparation BPs in wheat bread technology were analyzed. To reduce microbial contamination before the use as wheat bread ingredients, two treatments for the by-products were applied: fermentation with the *Lacticaseibacillus casei* LUHS210 strain and ultrasonication at 37 kHz. Furthermore, the wheat bread samples were prepared with the addition of different quantities (5, 10, 15, and 20%) of fermented and ultrasonicated by-products, and quality as well as safety parameters of the wheat bread were evaluated.

## Materials and Methods

### Plant Drink Preparation By-Products and Their Preparation for Bread Production

Plant-based drinks production BPs (AP, almond drink preparation by-product; OP, oat drink preparation by-product; CP, coconut drink preparation by-product) were obtained from a European company in 2019, producing plant-based drinks. The chemical composition of AP, CP, and OP (not dehydrated) is given in Table 1. *Moisture content* was determined by drying the samples to constant weight (ISO 712, 2009). *Ash content* was determined by calcinations at 900°C (ICC 104/1, 1990). *Dietary fibers* were determined according AOAC 991.43 method (AOAC, 1991). Kjeldahl method was used to determine *total proteins* (ISO 20483, 2013). *The total lipid content* was determined by extraction in the Soxhlet apparatus (“Boeco,” Germany) with hexane technical grade (Thermo Fisher Scientific, United States) (ICC 136, 1984). *Carbohydrates content* was calculated by the following formula: 100 − [weight in grams (protein + fat + water + ash) in 100 g of sample].

**TABLE 1:** Chemical composition of almond, coconut, and oat drinks preparation by-products (not dehydrated).

Sample	Moisture, %	Ash, %	Carbohydrates*, %	Dietary fibers, %	Protein, %	Lipid, %
AP	62.2 ± 0.2	6.7 ± 0.1	6.89 ± 0.91	8.31 ± 0.51	5.7 ± 0.5	10.2 ± 0.6
CP	55.0 ± 0.2	0.75 ± 0.04	28.14 ± 1.04	11.01 ± 0.41	4.9 ± 0.6	0.2 ± 0.02
OP	60.6 ± 0.1	2.28 ± 0.10	10.83 ± 0.81	10.09 ± 0.84	12.4 ± 0.9	3.8 ± 0.3

AP, almond drink preparation by-product; OP, oat drink preparation by-product; CP, coconut drink preparation by-product; *Carbohydrate is calculated by difference. Data expressed as a mean value (*n* = 3) ± SD; SD, standard deviation.

To disintegrate the by-products, all AP, CP, and OP samples were treated with a low-frequency (18 kHz) ultrasonic device (4.5 kW power, 50 μm amplitude, 340 W/cm^2^ intensity) for 2 min at 60°C at the Fraunhofer Institute UMSICHT (Germany). The BPs were stored in an airtight container at −18°C until further analysis.

#### Preparation of LAB Strain

*Lacticaseibacillus casei* LUHS210 was obtained from the Department of Food Safety and Quality at the Lithuanian University of Health Sciences (Kaunas, Lithuania) where it was stored at −80°C (Microbank system, Pro-Lab Diagnostics, Birkenhead, United Kingdom) until the use. From previous studies, it was known that the LUS210 strain possesses antimicrobial activities against various pathogenic and opportunistic strains (Bartkiene et al., 2019). Before the experiment *L. casei* LUHS210 was grown in Man-Rogosa-Sharpe (MRS) broth (CM 0359, Oxoid Ltd., Basingstoke, United Kingdom) at 30 ± 2°C for 48 h. Two percent of the MRS solution (v/v) in which the strain was multiplied was inoculated into fresh medium and propagated for 18 h. The multiplied LAB strain was further used for the fermentation of BPs.

#### Preparation of Fermented BPs

The BPs, water, and a suspension of the LUHS210 strain (3% from dry matter of the by-product mass) containing 8.9 log_10_ CFU/mL were fermented at 30 ± 2°C for 48 h. For 100 g of processed by-product, 60 mL of water was used. The final moisture content of the processed by-products was, on average, 60%. The moisture content was determined according to method described in ICC 109/01, 2020.

#### Preparation of Ultrasonicated BPs

Ultrasonication of BPs was performed at a frequency of 37 kHz using a 100 W power level. The equipment employed was a ultrasonication processor (PROCLEAN 3.0DSP, Ulsonix, Berlin, Germany). Each 250-g sample of by-product was processed for 30 min. Before ultrasonication, for 100 g of processed by-product, 60 mL of water was used.

### Acidity Characteristics of the Fermented By-Products

Total titratable acidity (TTA) and pH were measured only in not dehydrated fermented BPs.

By-products sample (5 g) was homogenized with 50 mL of distilled water and the pH was measured using a pH electrode (PP-15; Sartorius, Göttingen, Germany). TTA was determined in a10 mL of sample homogenized with 90 mL of distilled water, titrated with a volume (mL) of 0.1 mol/L NaOH to obtain a final pH value of 8.2, and expressed as Neiman degrees (°N). For the concentration evaluation of L-(+) and D-(−) lactic acid isomers, a specific Megazyme Assay Kit (Megazyme Int., Bray, Ireland) was used.

### Microbiological Analysis of Processing By-Products

The determination of LAB, total bacteria (TBC), enterobacteria (TEC), and mold/yeast (M/Y) counts in the by-products was performed according to Bartkiene et al. (2020b). Sterile MRS agar (CM0361; Oxoid) of 5 mm thickness was used for LAB growth on Petri dishes. The TBC was determined on plate count agar (CM0325; Oxoid). MacConkey (Oxoid Ltd., Basingstoke, United Kingdom) and tryptone bile X-glucuronide agar (Oxoid Ltd.) were used for the determination of the TEC (at 35–37°C for 18–24 h). M/Y counts were determined on chloramphenicol agar (CM0549; Oxoid).

### Bread Preparation

The wheat bread recipe consisted of 1 kg of flour (100%), 2% salt, 3% fresh compressed yeast, and 56% water (control bread). The dough was mixed (3 min at a low-speed regime and 8 min at a high-speed regime) in a mixer (Diosna SP25, Osnabrück, Germany), shaped, and proofed at 30°C and at 80% relative humidity for 45 min. Dough loaves of 350 g were formed and baked in a deck oven (MIWE Michael Wenz GmbH, Germany) at 220°C for 25 min. The wheat flour was substituted with 5, 10, 15, and 20% of fermented and ultrasonicated by-products. In total, 25 different wheat bread formulations were prepared and tested (bread without sourdough was used as the control; 24 formulations with 5, 10, 15, and 20% fermented and ultrasonicated AP, CP, and OP by-products were prepared).

### Dough and Bread Quality Evaluation

pH, TTA, color coordinates (L^∗^, a^∗^, b^∗^), and texture hardness were determined in dough with the addition of fermented BPs or ultrasonicated BPs. The samples were taken after the dough was mixed.

Bread samples were analyzed for TTA, moisture content, specific volume, crumb porosity, color coordinates (L^∗^, a^∗^, b^∗^), overall acceptability, bread shape coefficient, mass loss after baking, and acrylamide content. The analysis was performed 12 h after baking. In addition, bread crumb firmness during storage was evaluated.

#### Acidity Characteristics

Total titratable acidity of dough and bread were measured as described in section “Acidity Characteristics of the Fermented By-Products.” Dough pH was measured using pH electrode (PP-15; Sartorius, Göttingen, Germany) after dissolving 5 g of dough in 10 ml distilled water.

#### The Color Characteristics of Dough and Bread

The color characteristics of dough and bread were evaluated using a CIE L^∗^a^∗^b^∗^ (*L*^∗^ lightness; *a*^∗^ redness or −a^∗^ greenness; *b*^∗^ yellowness or −b^∗^ blueness) system (Chroma Meter CR-400, Konica Minolta, Japan; illuminant C, 10° observer).

#### Dough Texture Hardness

Dough texture hardness was determined using texture profile analysis (TPA) (Stevens-LFRA Texture Analyzer, Poland). Dough samples (50 g) were placed in a plastic measurement vessel (diameter of 25 mm and height of 50 mm) and hardness was fixed as a maximum compression force (20-mm diameter plunger, at a pre-test speed of 2 mm/s, test speed 10 mm/s, and penetration distance 10 mm). The samples were taken after the dough was mixed.

#### The Moisture Content of Bread

The moisture content of bread was established by drying the sample at 103 ± 2°C to constant weight.

#### Bread Crumb Porosity

Bread crumb porosity was ascertained according to the Lithuanian standard method (LST 1442, 1996).

#### Bread Volume

Bread volume was measured by the rapeseed displacement method (AACC, 2000).

#### Bread Specific Volume

Bread specific volume was calculated by measuring and dividing the volume and weight of the bread loaf.

#### The Bread Shape Coefficient

The bread shape coefficient was calculated by measuring and dividing the height and width of a bread slice.

#### The Overall Acceptability

The overall acceptability of each bread loaf was evaluated according to ISO 6658 (2017) using a 100 mm hedonic line scale ranging from 100 (extremely like) to 0 (extremely dislike). The sensory assessment was performed by 30 semi-trained panelists (16 women and 14 men aged 20–25 years). Before analysis, bread samples were sliced to 1.0 × 1.0 sub-samples and placed in plastic containers coded with three-digit codes and served to the evaluators.

#### Bread Crumb Firmness During Storage

Bread crumb firmness during storage was determined as the maximum compression force using texture profile analysis (TPA) (Stevens-LFRA Texture Analyzer, Poland). Bread was immediately sliced (thickness 6 mm), and crumb hardness was fixed as a maximum compression force (60% compression, 10-mm diameter plunger, compression rate of 2 mm/s).

#### The Quantitative Analysis of Acrylamide

The quantitative analysis of acrylamide was performed by liquid chromatography tandem mass spectrometry (LC-MS/MS) using a Waters Alliance HPLC system 2695 coupled to a Micromass Premier XE mass spectrometer (Micromass, Watford, United Kingdom), as described by Bartkiene et al. (2013b). The separation of acrylamide was achieved with a Luna 3 μm HILIC dC_18_ column (50 × 2.00 mm i.d., 3 μm; Phenomenex, Macclesfield, United Kingdom). The conditions for detection by MS/MS were as follows: ionization was performed using electrospray in the positive mode (source temperature of 120°C, desolvation temperature of 400°C; cone gas flow of 25 L h^–1^, desolvation gas flow of 600 L/h). The acrylamide concentrations in the selective samples were quantified by the internal standard (^13^C_3_-acrylamide) method.

### Statistical Analysis

Analyses are based on three independent fermentation and baking trials. Results were expressed as the mean ± standard deviation (SD). To evaluate the effects of the different types of by-products, their different treatments, and the quantity of additives used on bread quality parameters, the data were analyzed by multivariate analysis of variance (ANOVA) and Tukey’s honestly significant difference (HSD) procedure as *post-hoc* tests. A linear Pearson’s correlation was used to quantify the strength of the relationship between the variables (0.00–0.19, very weak; 0.20–0.39, weak; 0.40–0.59, moderate; 0.60–0.79, strong; 0.80–1.0, very strong). The correlation coefficients were calculated using the statistical package SPSS for Windows (v15.0, SPSS, Chicago, IL, United States). The results were recognized as statistically significant at *p* ≤ 0.05.

## Results

### Acidity and Microbiological Parameters of By-Products Fermented With LUHS210 Strain

**FIGURE 1:**
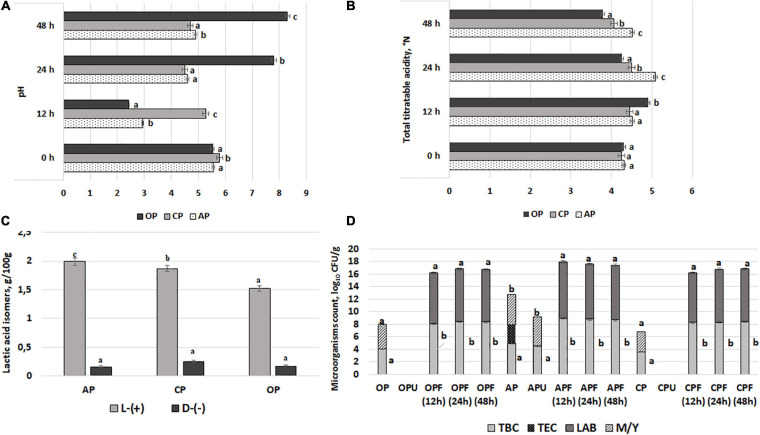
Acidity parameters [(**A**) – pH; (**B**) – total titratable acidity; (**C**) – L-(+) and (**D**)-(–) lactic acid concentration] of 12, 24, and 48 h fermented with LUHS210 strain by-products (AP, almond drink preparation by-product; OP, oat drink preparation by-product; CP, coconut drink preparation by-product). Microbiological parameters **(D)** of fermented with LUHS210 strain and ultrasonicated by-products (F, by-products fermented for 12, 24, and 48 h; U, ultrasonicated by-products; TBC, total bacteria count; TEC, total enterobacteria count; LAB, lactic acid bacteria; M/Y, mold/yeast). Data expressed as a mean value [(*n* = 3) ± SD]. ^*a*− *c*^ Means with different letters are significantly different (*p* < 0.05).

The pH, TTA, L-(+) and D-(−)-lactic acid concentration, and microbiological parameters of AP, CP, and OP by-products are shown in Figures 1A–D, respectively. The lowest AP and OP pH values after 12 h of fermentation were found (2.94 and 2.41, respectively). However, the lowest CP pH after 24 h of fermentation was obtained (4.5). The multivariate analysis of variance showed that both acidity parameters (pH and TTA) were significantly (*p* = 0.0001) affected by the fermentable substrate, the duration of fermentation, and the interaction of the analyzed factors (substrate × duration). When comparing the L-(+)/D-(−) lactic acid ratio, the highest ratio (12.4) in AP samples was found.

Total bacteria, TEC, LAB, and M/Y counts in samples after 12, 24, and 48 h of fermentation and ultrasonication are shown in Figure 1D. All of the initial by-product samples showed M/Y contamination, while enterobacteria were found in AP samples (3.08 log_10_ CFU/g). At the same time, samples that were ultrasonicated at 37 kHz did not contain enterobacteria, but the presence of M/Y was established in AP samples (4.61 log_10_ CFU/g). A comparison of fermented samples showed that none of these samples contained enterobacteria and M/Y, while the LAB count after 12 h of fermentation ranged from 7.90 to 8.95 log_10_ CFU/g (in CP and AP, respectively), the LAB count after 24 h of fermentation ranged from 8.38 to 8.75 log_10_ CFU/g (in OP and AP, respectively), and the LAB count after 48 h of fermentation ranged from 8.32 to 8.67 log_10_ CFU/g (in OP and AP, respectively). A moderate negative correlation between the pH values of the by-products and TBC was established (*r* = −0.4103), as well as a weak negative correlation (*r* = −0.1082) between the pH values of the by-products and the LAB count.

Finally, after summarizing the acidity and microbiological parameters of the fermented samples, for the wheat bread preparation, 12-h-fermented AP and OP samples (pH values of 2.94 and 2.41, respectively; in both samples, TEC and M/Y were not found) and 24-h-fermented CP samples (pH value of 4.50; TEC and M/Y were not found) were selected.

### Influence of the Fermented With the LUHS210 Strain and Ultrasonicated Almond, Coconut, and Oat By-Products on Dough Quality

The quality parameters (pH, TTA, color coordinates, and texture hardness) of dough, prepared with 5, 10, 15, and 20% of fermented and ultrasonicated AP, CP, and OP, are shown in Table 2. In the comparison of pH values of the control dough (without BPs) and the doughs with BPs, all the doughs prepared with fermented AP showed lower pH than the control dough (on average, 3.4% lower). However, fermented CP reduced the dough pH by 20%. Other dough samples prepared with lower fermented CP contents had higher pH values compared to the control dough (3.1% higher on average). Doughs prepared with 5, 15, and 20% of fermented OP showed reduced pH (by 5.2% on average) compared with the control dough. In comparison with the control doughs and the doughs prepared with ultrasonicated by-products, 5, 10, and 15% of AP and 10% and 15% of OP ultrasonicated by-products increased the dough pH; however, doughs prepared with 20% of ultrasonicated AP and doughs prepared with 5, 10, and 15% of ultrasonicated CP showed lower pH values compared to the control dough. Multivariate analysis of variance showed that the dough pH was significantly influenced by the by-product used (*p* = 0.043), the treatment applied for the by-product (*p* = 0.007), the quantity of by-product used (*p* ≤ 0.0001), and the interaction of the analyzed factors [by-product × treatment (*p* ≤ 0.0001); by-product × quantity (*p* = 0.003); by-product × quantity × treatment (*p* ≤ 0.0001)] was significant, except interaction of by-product quantity × treatment. No correlation was established between the dough pH and the TTA; the lowest TTA values of the dough samples prepared with CP and OP (fermented and ultrasonicated) were found (81.5% lower on average compared with the control dough). Dough samples with 5% fermented AP and with 15% and 20% ultrasonicated AP by-products had, on average, 17.3% higher TTA compared with the control dough. The dough TTA was significantly influenced by the by-product used (*p* ≤ 0.0001), and the interaction of the analyzed factors [by-product × treatment (*p* ≤ 0.0001); treatment × quantity (*p* = 0.004); by-product × quantity × treatment (*p* ≤ 0.0001)] was significant.

**TABLE 2:** Influence of the fermented with LUHS210 strain and ultrasonicated almond, coconut, and oat drinks preparation by-products on dough quality parameters.

Dough samples			pH	Color coordinates			Texture, mJ	TTA, °N
				a*	b*	L*		
Control			5.45 ± 0.07^*d*^	0.50 ± 0.04^*a*^	23.76 ± 0.23	91.08 ± 2.50	0.27 ± 0.01^*b*^	8.00 ± 0.09^*d*^
AP	Fermented	5%	5.26 ± 0.09^*c*^	0.94 ± 0.15 ^*b*^	24.00 ± 1.54	88.74 ± 3.48	0.27 ± 0.01^*b*^	9.67 ± 0.08^*d*^
		10%	5.30 ± 0.04^*c*^	0.80 ± 0.13^*b*^	23.65 ± 0.40	91.00 ± 1.41	0.27 ± 0.03^*b*^	9.00 ± 1.00^*d*^
		15%	5.09 ± 0.07^*b*^	1.10 ± 0.26^*b*^	23.65 ± 0.10	89.49 ± 2.98	0.20 ± 0.01^*a*^	8.00 ± 0.00^*d*^
		20%	5.34 ± 0.04^*c*^	1.01 ± 0.24^*b*^	23.42 ± 0.42	90.59 ± 1.91	0.17 ± 0.01^*a*^	8.00 ± 1.00^*d*^
	Ultrasonicated	5%	5.82 ± 0.03e	0.92 ± 0.19b	24.17 ± 0.76	87.97 ± 4.22	0.27 ± 0.02b	8.67 ± 0.58d
		10%	5.57 ± 0.01d	0.71 ± 0.18b	22.95 ± 1.34	88.92 ± 3.47	0.27 ± 0.02b	8.67 ± 0.58d
		15%	5.87 ± 0.0^*e*^	0.84 ± 0.16^*b*^	23.22 ± 0.54	89.38 ± 2.66	0.27 ± 0.01^*b*^	9.67 ± 0.58^*d*^
		20%	5.28 ± 0.04c	0.71 ± 0.12b	23.36 ± 0.73	89.65 ± 2.10	0.27 ± 0.02b	9.67 ± 0.58d
CP	Fermented	5%	5.61 ± 0.02^*d*^	0.51 ± 0.09^*a*^	23.76 ± 0.23	94.41 ± 3.91	0.27 ± 0.03^*b*^	0.77 ± 0.07^*a*^
		10%	5.69 ± 0.06^*d*^	0.49 ± 0.05^*a*^	24.06 ± 1.00	90.01 ± 1.46	0.20 ± 0.02^*a*^	1.40 ± 0.07^*b*^
		15%	5.56 ± 0.02^*d*^	0.73 ± 0.09^*b*^	24.81 ± 1.50	87.68 ± 3.64	0.17 ± 0.01^*a*^	1.33 ± 0.05^*b*^
		20%	5.25 ± 0.06^*c*^	0.76 ± 0.06^*b*^	21.47 ± 1.38	90.40 ± 0.61	0.15 ± 0.03^*a*^	0.93 ± 0.04^*a*^
	Ultrasonicated	5%	5.24 ± 0.03^*c*^	0.58 ± 0.08^*a*^	23.59 ± 1.16	87.18 ± 3.89	0.30 ± 0.03^*b*^	0.63 ± 0.06^*a*^
		10%	5.21 ± 0.02^*c*^	0.81 ± 0.11^*b*^	23.24 ± 1.33	87.44 ± 4.84	0.27 ± 0.03^*b*^	0.67 ± 0.05^*a*^
		15%	4.80 ± 0.05^*a*^	0.77 ± 0.15^*b*^	22.90 ± 1.35	88.71 ± 2.56	0.40 ± 0.01^*d*^	0.77 ± 0.06^*a*^
		20%	5.38 ± 0.07^*cd*^	0.92 ± 0.18^*b*^	23.31 ± 0.74	91.58 ± 1.30	0.40 ± 0.02^*d*^	0.93 ± 0.07^*a*^
OP	Fermented	5%	5.39 ± 0.05^*cd*^	1.68 ± 0.15^*c*^	25.74 ± 2.51	91.71 ± 1.62	0.30 ± 0.03^*b*^	2.50 ± 0.02^*c*^
		10%	5.69 ± 0.05^*d*^	2.71 ± 0.11^*d*^	25.26 ± 2.30	87.82 ± 3.01	0.27 ± 0.01^*b*^	2.40 ± 0.03^*c*^
		15%	5.12 ± 0.05^*b*^	2.86 ± 0.18^*de*^	26.08 ± 2.73	88.09 ± 3.92	0.31 ± 0.04^*b*^	2.20 ± 0.01^*c*^
		20%	4.99 ± 0.02^*b*^	3.22 ± 0.19^*e*^	27.00 ± 4.81	89.40 ± 2.94	0.31 ± 0.04^*b*^	2.10 ± 0.10^*c*^
	Ultrasonicated	5%	5.50 ± 0.02^*d*^	1.65 ± 0.21^*c*^	25.57 ± 2.16	87.97 ± 3.89	0.24 ± 0.03^*b*^	2.10 ± 0.10^*c*^
		10%	5.76 ± 0.05^*e*^	1.92 ± 0.12^*c*^	25.02 ± 2.53	88.47 ± 2.3	0.27 ± 0.02^*b*^	2.10 ± 0.03^*c*^
		15%	5.66 ± 0.03^*d*^	2.67 ± 0.15^*d*^	25.05 ± 2.25	89.91 ± 2.8	0.24 ± 0.03^*b*^	1.60 ± 0.04^*b*^
		20%	5.43 ± 0.08^*d*^	2.36 ± 0.14^*d*^	25.27 ± 2.81	90.23 ± 2.17	0.27 ± 0.01^*b*^	1.30 ± 0.05^*b*^

AP, almond drink preparation by-product; OP, oat drink preparation by-product; CP, coconut drink preparation by-product. Control – bread produced without by-products; (5, 10, 15, 20%) – dough produced with 5, 10, 15, 20%, respectively, of by-products. L*, lightness; a*, redness (−a* greenness); b*, yellowness (−b* blueness). Data expressed as a mean value (*n* = 3) ± SD; SD, standard deviation.^a–*e*^ Means with different letters in columns are significantly different (*p* < 0.05).

In the comparison of the color coordinates of the doughs (L^∗^, lightness; a^∗^, redness; b^∗^, yellowness), in most of the cases, by-product addition increased the a^∗^ coordinate of the dough compared with the control dough, which was prepared without by-products. In compare all the groups, the highest a^∗^ of dough samples prepared with OP was found (on average, by 80.9 and 76.7% higher, of the doughs prepared with fermented and ultrasonicated by-products, respectively, compare with control).

When comparing the texture hardness of the doughs, it was established that by increasing the content of the fermented AP (at concentrations of 15% and 20%) and fermented CP (at concentrations of 10, 15, and 20%), the hardness of the doughs was reduced (AP doughs by 25.9 and 37.0%, respectively, and CP doughs by 25.9, 37.0, and 44.4%, respectively, compared with the control dough). Conversely, by the addition of ultrasonicated CP additives at concentrations of 15% and 20%, the hardness of the doughs increased significantly (by 32.5% on average compared with the control).

### Influence of the Fermented With the LUHS210 Strain and Ultrasonicated By-Products on Wheat Bread Quality Parameters

The quality parameters (specific volume, porosity, moisture content, TTA, overall acceptability, bread shape coefficient, and mass loss after baking) of wheat bread prepared with fermented and ultrasonicated BPs are shown in Table 3. In compare wheat bread specific volume, significant differences between the control breads and breads prepared with fermented, as well as ultrasonicated AP by-products were not found. In compare samples prepared with CP, it was found that fermented CP and ultrasonicated CP addition was not significant on bread specific volume, except samples with 20% of ultrasonicated CP, which specific volume was found lower (on average, by 10%), compare with controls. In compare control bread and bread prepared with OP, it was found that 5% of ultrasonicated OP significantly increased bread specific volume (by 14.4%). Between bread specific volume and porosity strong positive correlation was established (*r* = 0.7747). By increasing by-products content in bread formulations, moisture content of the breads was increased, and between breads TTA and moisture content moderate positive correlation was found (*r* = 0.4948). The TTA of the breads was increased, by increasing content of the fermented by-products in bread formulations. Also, by increasing bread specific volume and porosity, moisture content of the samples showed tendency to reduce, and negative moderate correlations between the bread specific volume and porosity with the moisture content were found (*r* = −0.4212 and *r* = −0.5467, respectively). In compare bread samples mass loss after baking, 5% of the fermented and ultrasonicated AP, 5 and 10% of the ultrasonicated AP, and 15% of the ultrasonicated CP reduces breads mass loss after baking, compare with control breads, and between the moisture content and mass loss after baking negative moderate correlation was found (*r* = −0.4323). The highest overall acceptability of the breads, prepared with 20% of fermented AP, 15% of fermented OP, as well as 15% of ultrasonicated OP was established. However, it should be mentioned that the specific volume and porosity has a low influence on overall acceptability of the breads, as above mentioned parameters showed very weak correlation with overall acceptability (*r* = 0.1210 and *r* = 0.1413, respectively). In all the cases OP (fermented and ultrasonicated) reduce bread shape coefficient, compare with control breads. Also, lower bread shape coefficient of the samples, prepared with 5 and 10% of fermented and 5% of ultrasonicated AP by-products, as well as with 5% of fermented and 15% of ultrasonicated CP was found. Finally, 20% of fermented AP, 15% of fermented OP, and 15% of ultrasonicated OP could increase overall acceptability of the wheat bread; however, further analyzed characteristics (bread staling process and acrylamide content) are very important, as it can lead to future consumers choice of the proposed product.

**TABLE 3:** Influence of the fermented with LUHS210 strain and ultrasonicated almond, coconut, and oat drinks preparation by-products on wheat bread quality parameters.

Bread samples			Specific volume, cm^3^/g	Porosity, %	Moisture content, %	TTA, °N	Overall acceptability	Bread shape coefficient	Mass loss after baking, %
Control			3.33 ± 0.29bc	74.56 ± 0.33	24.60 ± 0.72	1.5 ± 0.1a	102.1 ± 7.64g	2.3 ± 0.1	11.36 ± 0.38^*c*^
AP	Fermented	5%	3.44 ± 0.13^*bc*^	74.43 ± 0.20	31.20 ± 0.60	2.8 ± 0.1^*c*^	75.3 ± 6.53^*f*^	2.0 ± 0.1	10.81 ± 0.13^*b*^
		10%	3.28 ± 0.09^*b*^	74.34 ± 0.25	33.40 ± 1.91	3.9 ± 0.2e	86.1 ± 4.51f	1.8 ± 0.2	12.40 ± 0.26d
		15%	3.42 ± 0.10^*bc*^	74.39 ± 0.21	33.53 ± 1.96	4.1 ± 0.2e	109.2 ± 4.00g	2.4 ± 0.1	11.54 ± 0.10c
		20%	3.33 ± 0.21^*bc*^	74.67 ± 0.21	34.47 ± 1.55	5.7 ± 0.2f	126.5 ± 3.51h	2.2 ± 0.1	12.76 ± 0.56e
	Ultrasonicated	5%	3.46 ± 0.14^*bc*^	74.63 ± 0.13	31.07 ± 0.99	1.7 ± 0.2^*a*^	63.3 ± 4.51^*d*^	1.9 ± 0.2	9.33 ± 0.76^*a*^
		10%	3.36 ± 0.07^*b*^	74.37 ± 0.21	32.13 ± 0.45	1.8 ± 0.1^*a*^	48.1 ± 2.52^*c*^	2.2 ± 01	10.43 ± 0.26^*ab*^
		15%	3.33 ± 0.02^*b*^	74.88 ± 0.38	32.87 ± 0.64	1.8 ± 0.1^*a*^	37.3 ± 3.51^*b*^	2.2 ± 0.1	11.67 ± 0.13^*c*^
		20%	3.22 ± 0.14^*ab*^	74.53 ± 0.11	33.13 ± 0.42	1.8 ± 0.1a	18.6 ± 4.00a	2.2 ± 0.2	11.37 ± 0.26c
CP	Fermented	5%	3.22 ± 0.15^*ab*^	74.56 ± 0.10	23.40 ± 0.92	1.7 ± 0.1^*a*^	110.5 ± 5.5^*g*^	1.8 ± 0.3	12.90 ± 0.44^*e*^
		10%	3.50 ± 0.09^*c*^	76.09 ± 0.15	23.73 ± 0.92	2.0 ± 0.2^*a*^	100.2 ± 7.0^*g*^	2.5 ± 0.1	15.17 ± 0.81^*g*^
		15%	3.35 ± 0.04^*b*^	74.64 ± 0.21	26.07 ± 0.61	2.3 ± 0.1^*a*^	85.4 ± 7.7^*f*^	2.3 ± 0.1	15.47 ± 0.36^*g*^
		20%	3.31 ± 0.08^*b*^	74.59 ± 0.09	26.93 ± 0.90	3.5 ± 0.1^*d*^	70.2 ± 5.6f	2.2 ± 0.1	14.85 ± 0.76^*g*^
	Ultrasonicated	5%	3.31 ± 0.11^*b*^	74.72 ± 0.23	23.13 ± 1.16	1.7 ± 0.2^*a*^	83.3 ± 7.5^*f*^	2.4 ± 0.3	12.06 ± 0.12^*cd*^
		10%	3.23 ± 0.10^*b*^	74.63 ± 0.31	24.20 ± 0.53	1.9 ± 0.2^*ab*^	61.3 ± 4.3^*d*^	2.1 ± 0.1	11.69 ± 0.33^*c*^
		15%	3.18 ± 0.13^*ab*^	74.27 ± 0.38	31.73 ± 1.10	1.7 ± 0.1^*a*^	56.0 ± 4.5^*c*^	1.9 ± 0.2	9.85 ± 0.36^*a*^
		20%	3.00 ± 0.10^*a*^	72.47 ± 0.17	33.07 ± 1.09	1.7 ± 0.1^*a*^	45.7 ± 4.3^*c*^	2.1 ± 0.2	12.05 ± 0.50^*c*^
OP	Fermented	5%	3.71 ± 0.17^*cd*^	75.18 ± 0.23	23.48 ± 0.47	1.7 ± 0.2^*a*^	48.53 ± 14.52^*bcd*^	1.8 ± 0.1	13.90 ± 0.01^*f*^
		10%	3.60 ± 0.12^*c*^	75.05 ± 0.12	24.40 ± 0.61	1.9 ± 0.2^*a*^	71.88 ± 9.55^*f*^	1.8 ± 0.1	13.40 ± 0.05^*f*^
		15%	3.53 ± 0.07^*c*^	75.10 ± 0.10	30.82 ± 0.55	2.0 ± 0.1^*a*^	135.65 ± 12.64^*h*^	1.8 ± 0.1	13.50 ± 0.03^*f*^
		20%	3.42 ± 0.11^*bc*^	74.66 ± 0.30	31.85 ± 1.00	2.0 ± 0.2^*a*^	93.14 ± 13.37f^*g*^	1.8 ± 0.1	13.50 ± 0.02^*f*^
	Ultrasonicated	5%	3.81 ± 0.05^*d*^	75.89 ± 0.21	23.48 ± 0.47	1.7 ± 0.2^*a*^	52.05 ± 14.51^*bcd*^	1.7 ± 0.1	12.80 ± 0.01^*e*^
		10%	3.62 ± 0.19^*cd*^	75.12 ± 0.14	24.40 ± 0.61	1.5 ± 0.3^*a*^	88.42 ± 11.34f^*g*^	1.6 ± 0.2	12.80 ± 0.01^*e*^
		15%	3.41 ± 0.10^*bc*^	74.62 ± 0.25	29.82 ± 0.55	1.7 ± 0.1^*a*^	123.57 ± 14.35^*h*^	1.9 ± 0.1	12.70 ± 0.02^*e*^
		20%	3.33 ± 0.14^*b*^	74.52 ± 0.15	30.85 ± 1.00	1.7 ± 0.1^*a*^	78.32 ± 10.67^*f*^	2.0 ± 0.1	12.70 ± 0.01^*e*^

*AP, almond drink preparation by-product; OP, oat drink preparation by-product; CP, coconut drink preparation by-product. Control – bread produced without by-products; (5, 10, 15, 20%) – bread produced with 5, 10, 15, 20%, respectively, of by-products. Data expressed as a mean value (*n* = 3) ± SD; SD, standard deviation.^a–*h*^ Means with different letters in columns are significantly different (*p* < 0.05).*

### Influence of the Fermented With LUHS210 Strain and Ultrasonicated By-Products on Wheat Bread Texture Hardness During the Storage

**FIGURE 2:**
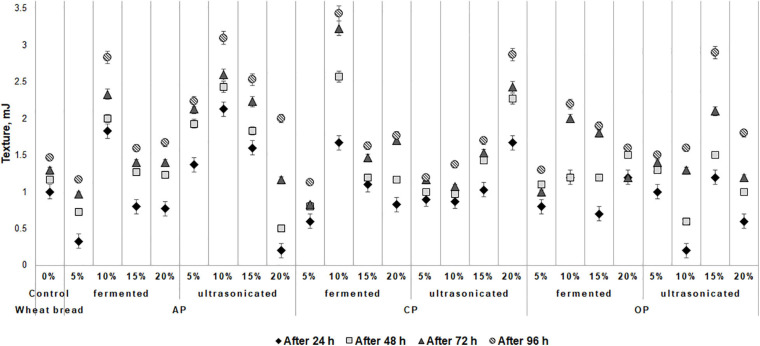
Influence of the fermented with LUHS210 strain and ultrasonicated by-products (AP, almond drink preparation by-product; OP, oat drink preparation by-product; CP, coconut drink preparation by-product) on wheat bread texture during the storage [Control – bread produced without by-products; (5, 10, 15, 20%) – bread produced with 5, 10, 15, 20%, respectively, of by-products. Data expressed as a mean value (*n* = 3) ± SD; SD, standard deviation].

The changes of wheat bread texture hardness during the storage are shown in Figure 2. The lowest bread hardness after 24 h of storage was found of the samples, prepared with addition of 5% of fermented and 20% of ultrasonicated AP (0.33 and 0.20 mJ, respectively) and with 10% of ultrasonicated OP (0.20 mJ). In compare all the breads prepared with by-products with control, after 24 h of storage, lower hardness of the breads, prepared with 15% of fermented AP, with 5% of fermented and ultrasonicated CP, with 5 and 15% of fermented and 20% of ultrasonicated OP was found. After 48 h of storage, lower hardness, in compare with control breads, of the breads, prepared with 5% of fermented and 20% of ultrasonicated AP (0.73 and 0.50 mJ, respectively), with 5% of fermented and 5 and 10% of ultrasonicated CP (0.80, 1.00, and 0.97 mJ, respectively), and with 10 and 20% of ultrasonicated OP (0.60 and 1.00 mJ, respectively) by-products was found. Similar tendencies after 72 h of storage were established, however, after 96 h of storage, the lowest hardness (lower than that control bread) of the breads prepared with 5% of fermented AP, CP, and OP (1.17, 1.13, and 1.30 mJ, respectively) and with 5% of ultrasonicated CP (1.2 mJ) was found. Weak correlation between the bread moisture content and texture hardness after 96 h of storage was found (*r* = 0.3399), however, with other analyzed bread parameters correlations with texture hardness were not established. Also, a weak correlation between the dough pH and bread texture hardness after 96 h of storage was found (*r* = 0.3849).

### Influence of the Fermented With the LUHS210 Strain and Ultrasonicated By-Products on Wheat Bread Crust and Crumb Color Coordinates and Acrylamide Formation

**FIGURE 3:**
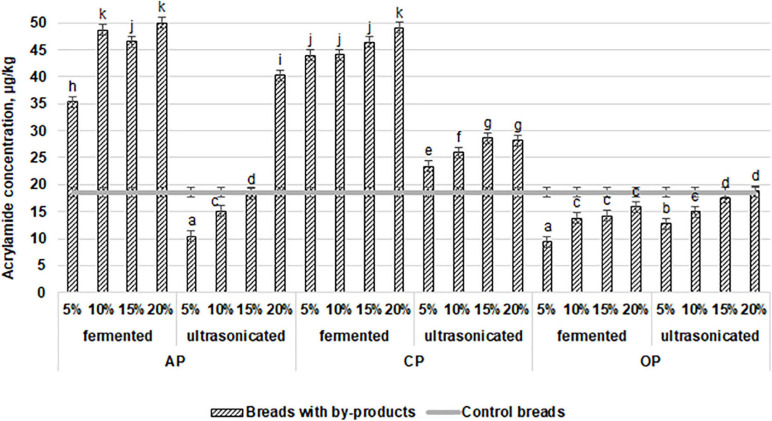
Influence of the fermented with LUHS210 strain and ultrasonicated by-products (AP, almond drink preparation by-product; OP, oat drink preparation by-product; CP, coconut drink preparation by-product) on acrylamide concentration (μg/kg) in wheat bread. Data expressed as a mean value (*n* = 3) ± SD; SD, standard deviation; ^*a*− *k*^ Means with different letters are significantly different (*p* < 0.05).

Wheat bread crust and crumb color coordinates are shown in Table 4. In a comparison of the averages of the different wheat bread groups (prepared with fermented and ultrasonicated AP, CP, and OP and crust and crumb color coordinates, significant differences between the crust and crumb color coordinates were not found. However, compared with the control group, significantly higher bread crumb redness (a^∗^) of all breads prepared with the addition of by-products was found (33.8, 31.9, 40.5, 30.8, 58.5, and 64.3% higher, in prepared with fermented and ultrasonicated AP, CP, and OP, respectively). In compare acrylamide concentration in wheat bread samples, in all the bread groups the same tendency was established that by the increasing the tested by-products concentration in wheat bread formula, acrylamide concentration was increased, and the highest acrylamide concentration in breads, prepared with the addition of fermented AP and CP was found [on average, by 2.5 times higher, compare with control bread (Figure 3)]. However, ultrasonicated AP and CP showed lower influence on acrylamide formation, and in bread groups, prepared with 5 and 10% of ultrasonicated AP, significantly lower (on average, by 44.1 and 18.8%, respectively) acrylamide concentration was found, compare with control breads. However, addition of 20% of ultrasonicated AP increases acrylamide concentration, on average, by 53.8%, compare with control breads. In breads, prepared with 5, 10, 15, and 20% ultrasonicated CP, acrylamide concentration was, on average, by 20.5, 28.2, 34.9, and 34.0%, respectively, higher, compare to control samples. The lowest acrylamide concentrations in breads, prepared with OP by-products were found (lower then that in control breads, except breads prepared with 15 and 20% of ultrasonicated OP). Also, between the acrylamide concentration in bread and crust color coordinates correlations were found: very weak negative with a^∗^ coordinate (*r* = −0.1206), moderate negative with yellowness (b^∗^) coordinate (*r* = −0.4538), and weak negative with lightness (L^∗^) coordinate (*r* = −0.4538). The main acrylamide content usually is in bread crust, however, in this study between acrylamide concentration in bread and crumb color coordinates, also, correlations were found: moderate negative with a^∗^ coordinate (*r* = −0.5283), weak negative with b^∗^ coordinate (*r* = −0.2479), and weak positive with L^∗^ coordinate (*r* = −0.3621). As well as multivariate analysis of variance showed that the different type of by-products, different their treatment, the quantity of used additives and above mentioned factors interaction was significant on acrylamide concentration in bread (*p* = 0.0001).

**TABLE 4:** Influence of the fermented with LUHS210 strain and ultrasonicated almond, coconut, and oat drinks preparation by-products on wheat bread crust and crumb color coordinates.

Bread samples			Crust color coordinates			Crumb color coordinates		
			a*	b*	L*	a*	b*	L*
Control			13.74 ± 0.31	20.64 ± 0.38	44.58 ± 3.82	0.94 ± 0.06a	24.65 ± 1.13	74.69 ± 3.17
AP	Fermented	5%	15.35 ± 1.32	27.08 ± 2.37	49.27 ± 3.70	1.09 ± 0.08^*b*^	25.59 ± 1.34	78.11 ± 4.02
		10%	14.98 ± 1.43	28.66 ± 2.67	54.33 ± 3.37	1.31 ± 0.15^*bc*^	25.50 ± 1.21	76.66 ± 4.87
		15%	12.39 ± 1.84	19.33 ± 1.69	43.36 ± 3.82	1.56 ± 0.06^*d*^	25.84 ± 1.21	76.73 ± 3.36
		20%	11.26 ± 0.84	20.80 ± 1.14	48.59 ± 2.71	1.73 ± 0.08^*e*^	25.10 ± 1.25	74.77 ± 5.60
	Ultrasonicated	5%	13.55 ± 1.24	22.90 ± 1.92	48.27 ± 3.11	1.52 ± 0.06d	25.77 ± 1.17	76.60 ± 3.47
		10%	12.30 ± 1.41	23.31 ± 1.99	51.04 ± 2.92	1.44 ± 0.07^*cd*^	24.51 ± 1.36	74.78 ± 4.30
		15%	11.44 ± 0.13	19.31 ± 1.53	45.08 ± 2.63	1.36 ± 0.08^*c*^	24.67 ± 1.33	74.12 ± 3.57
		20%	12.21 ± 1.27	19.22 ± 0.80	44.71 ± 3.38	1.18 ± 0.10^*b*^	24.68 ± 1.25	75.68 ± 3.40
CP	Fermented	5%	13.74 ± 0.31	20.64 ± 1.38	44.58 ± 2.82	0.94 ± 0.06^*a*^	24.65 ± 1.13	74.69 ± 4.17
		10%	13.20 ± 0.57	17.74 ± 1.78	47.03 ± 2.15	1.11 ± 0.09^*b*^	24.69 ± 1.05	75.81 ± 4.39
		15%	10.16 ± 1.08	16.07 ± 1.05	43.06 ± 2.34	1.25 ± 0.10^*bc*^	23.63 ± 0.95	72.97 ± 3.47
		20%	11.00 ± 0.16	22.08 ± 1.11	55.32 ± 2.37	1.33 ± 0.07^*c*^	22.98 ± 1.28	73.42 ± 3.62
	Ultrasonicated	5%	12.00 ± 0.72	20.90 ± 1.68	49.04 ± 2.36	1.40 ± 0.10^*cd*^	24.85 ± 1.43	72.84 ± 3.38
		10%	14.62 ± 0.39	23.34 ± 1.65	48.47 ± 2.65	1.11 ± 0.08^*b*^	24.74 ± 1.22	75.19 ± 3.16
		15%	14.00 ± 0.98	21.99 ± 2.33	47.11 ± 3.90	0.97 ± 0.07^*ab*^	24.62 ± 1.19	75.36 ± 3.44
		20%	16.10 ± 0.39	27.34 ± 1.80	51.31 ± 2.56	1.95 ± 0.10^*e*^	24.41 ± 1.08	76.35 ± 4.21
OP	Fermented	5%	12.78 ± 0.50	28.05 ± 1.98	59.47 ± 3.75	1.86 ± 0.11^*e*^	24.93 ± 1.52	73.90 ± 3.09
		10%	11.95 ± 0.21	25.08 ± 1.40	56.07 ± 2.42	2.58 ± 0.23^*g*^	24.47 ± 2.40	72.18 ± 3.28
		15%	12.79 ± 0.83	25.48 ± 1.59	49.16 ± 2.45	2.78 ± 0.12^*g*^	26.13 ± 1.96	73.71 ± 2.96
		20%	11.98 ± 0.62	23.30 ± 1.33	48.49 ± 3.08	1.86 ± 0.09^*e*^	24.93 ± 1.53	73.42 ± 3.08
	Ultrasonicated	5%	12.89 ± 0.45	25.43 ± 1.96	49.97 ± 2.74	2.20 ± 0.12^*f*^	25.28 ± 1.73	75.34 ± 3.10
		10%	14.48 ± 0.91	26.61 ± 1.67	51.77 ± 3.19	2.83 ± 0.11^*g*^	24.83 ± 0.63	71.85 ± 2.89
		15%	13.13 ± 0.53	28.63 ± 0.52	54.22 ± 2.08	2.75 ± 0.14^*g*^	24.85 ± 1.36	71.19 ± 4.53
		20%	13.46 ± 0.37	27.29 ± 1.34	53.77 ± 3.37	2.75 ± 0.16^*g*^	24.82 ± 1.49	69.59 ± 3.68

AP, almond drink preparation by-product; OP, oat drink preparation by-product; CP, coconut drink preparation by-product. Control – bread produced without by-products; (5, 10, 15, 20%) – bread produced with 5, 10, 15, 20%, respectively, of by-products. L*, lightness; a*, redness (−a* greenness); b*, yellowness (−b* blueness). Data expressed as a mean value (*n* = 3) ± SD; SD, standard deviation. ^*a*–*g*^ Means with different letters in columns are significantly different (*p* < 0.05).

## Discussion

In this study, the LUHS210 strain was used since it has a versatile carbohydrate metabolism (Bartkiene et al., 2019, 2020c). This strain was previously isolated from a spontaneous rye sourdough and the evaluation of LAB growth under acidic environments showed that *L. casei* LUHS210 had a good microbial viability at pH 2.5 – the concentration of viable cells after 2 h incubation at pH 2.5 was 8.36 ± 0.2 (CFU/mL) (Bartkiene et al., 2020c). The activity of the LAB culture elicits a pH reduction in the substrate during the fermentation process (Freire et al., 2015). Differences in pH values between AP, OP, and CP could be related to the different amounts of soluble protein and fermentable carbohydrates, which impact organic acid production and the acidification level (Juodeikiene et al., 2011). The pH in fermented CP was the lowest because it contained higher amount of carbohydrates compared to other BPs (Table 1). Also, pH could depend on amino acids, i.e., if acid is added to a solution containing the zwitterion, the carboxylate group captures a hydrogen (H^+^) ion, and the amino acid becomes positively charged (Chemistry LibreTexts, 2020). If base is added, ion removal of the H^+^ ion from the amino group of the zwitterion produces a negatively charged amino acid. In both circumstances, the amino acid acts to maintain the pH of the system – that is, to remove the added acid (H^+^) or base (OH^–^) from solution. Moreover, the alkaline-producing activities also occur during fermentation. The increase in pH during fermentation could be related with the higher protein content in substrate, proteolytic activities, and the release of ammonia by microorganisms involved in fermentation. Protein content in OP was the highest (Table 1) between other samples, therefore the pH in OP was higher. The similar pH tendency was also observed during soybeans and tamarind fermentation (Dakwa et al., 2005; Olagunju et al., 2018). During the fermentation of BPs, free -NH_3_ groups could be realized in the substrate, and in contact with organic acids, they could be neutralized, for this reason, an intensive smell of the ammonia was not observed in the final products.

As the main acidic nature metabolites of lacto fermentation, L(+) and D(−)-lactic acid isomers were also evaluated in this study. L-(+)-lactic acid was produced in a significantly higher amount than D-(−)-lactic acid among all fermented by-products. L- and D-lactate dehydrogenases are responsible for the production of lactic acid optical isomers. L-(+)-lactic acid is also synthesized in the human body, while D-(−)-lactic acid is a toxic compound and may cause acidosis when accumulated in the body (Bianchetti et al., 2018).

Changes in microbiological parameters of BPs occurred due to fact that ultrasonication and fermentation with LAB could affect the activity of microorganisms in different ways. Due to the wide range of inhibitory compounds (lactic and acetic acids, bacteriocins, acetoin, hydrogen peroxide, etc.) produced by LAB, as well as species of the *Lacticaseibacillus casei* group, the growth of pathogenic and opportunistic microorganisms could be limited in fermented substrates (Kim and Ndegwa, 2018).

When high intensity ultrasound is applied, the cavitation phenomenon increases cell membrane penetrability and induces the formation of free radicals in the cells of microorganisms (Ferrario et al., 2015). This leads to the deactivation of microbial cells. However, the inactivation level depends on the food matrix and the ultrasonication conditions (frequency, power, time). The inhibitory effect of ultrasonication alone and in combination with other techniques was reported in the current literature (Fan et al., 2019; Wang and Fan, 2019; Pagnossa et al., 2020; Yu et al., 2020).

After summarizing the acidity and microbiological parameters of the fermented samples, for the wheat bread preparation, 12-h-fermented AP and OP samples (pH values of 2.94 and 2.41, respectively; in both samples, TEC and M/Y were not found) and 24-h-fermented CP samples (pH value of 4.50; TEC and M/Y were not found) were selected.

In general, the addition of different fermented plant drink preparation BPs with lower pH could reduce the dough pH and affect the solubility of the dough components and the activity of the endogenous enzymes (Savkina et al., 2019). However, the amount of fermented BPs, which was added into the dough, was quite small, so the change in dough pH may not be significant when compared with control dough. Moreover, the additional dough fermentation was not applied in this case. As can be seen from the Table 2, the pH of dough after addition decreased or stay similar. When pH is assessed, the concentration of hydrogen ions that they release in an aqueous solution is determined. TTA shows the content of acid present, which does not necessarily relate to the concentration of the hydrogen ions. Therefore, pH and TTA values in Table 2 and Figure 1 could be inconsistent because LAB fermentation is a live biological system and consistency could not always be reach in this kind of systems. Moreover, the changes in the acidity levels of the tested doughs could be influenced by yeast-produced carbon dioxide (Balestra et al., 2015).

Dough color was affected by the color of the raw materials; therefore, the increase in the value of the a^∗^ coordinate of the doughs could be related to the redness-brownness color of AP, CP, and OP. However, a significant influence of the by-products on the b^∗^ and L^∗^ coordinates of the doughs was not established. Changes in the texture of the wheat dough, supplemented with plant drink preparation by-products, could be related to the higher content of fat, non-gluten proteins, and dietary fibers in AP, OP, and CP, which have an impact on gluten dilution and increased water absorption (Sudha et al., 2012; Cantatore et al., 2019). When fermented additives are used, wheat dough may become softer and less elastic due to proteolysis and microbial hydrolysis of starch (Aplevicz et al., 2013). Ultrasound could lead to the disintegration of compounds in plant drink preparation by-products, which affects the physical quality of the dough (Berezina et al., 2018). Therefore, due to physical and chemical changes induced by ultrasonication in CP, a higher hardness of the dough with ultrasonicated CP was found.

The reduced carbon dioxide gas holding capacity due to weakened gluten network influences the specific volume and bread shape coefficient of breads with plant drinks preparation BP (Cantatore et al., 2019). However, LAB activity in fermented products could positively affect composite bread quality parameters by increasing soluble protein content and reducing disulphide bonds in gluten network (Hadaegh et al., 2017). Despite studies on composite breads quality parameters, there is a little or no information available on the usage of fermented and ultrasonicated AP, CP, or OP for bread production. Oat flour or bran addition to wheat flour resulted in lower bread loaf volume; however, sourdough improved oat-wheat bread quality and sensory properties (Różyło et al., 2014). Reduction in specific volume and increase in moisture content in coconut-wheat composite breads were reported by Erminawati et al. (2017) and Awe et al. (2018). Same tendencies were obtained with fermented cashew kernel-wheat composite bread (Touzou et al., 2019). However, the acceptance of produced composite breads could be diverse. In this case, fermentation with LAB could give the additional taste and aroma to the produced bread and increase the acceptability score. In research of Kulasekar and Amritkumar (2018), fermented with LAB coconut residue-composite bread had higher overall acceptability compared to other tested breads, while (Touzou et al., 2019) reported that fermented cashew kernel-wheat composite bread was acceptable to consumer.

Interaction between starch and gluten protein, water migration and amylopectin retrogradation are assumed to play an important role in bread staling during storage (Barros et al., 2018). The ratio of water and starch may affect the staling rate (Kurek et al., 2017). AP, OP, and CP contain a high amount of dietary fiber with water-holding capacity and that could serve as additional source of water. However, a higher substitution level of fibers could alter rheological behavior and quality parameters of composite bread in different ways. Moreover, other components such as lipids, sugars or enzymes could also affect bread staling (Kurek et al., 2017). In this study, the reduced hardness of breads with 5% of fermented additives at the end of storage could be related with the increased acidity, protein solubility and formation of exopolysaccharides during fermentation, which may modify bread texture and delay staling process (Katina et al., 2006).

Consumer choice of bread is firstly influenced by the visual appearance of the product, especially the color of the golden crust or white crumbs (Hayta and Ertop, 2019). During baking, a high temperature in crumb is not reached; therefore, dough ingredients are mostly responsible for crumb color (Cantatore et al., 2019). Moreover, other dough components, such as water, sugars, amino acids, and pH, could affect bread color. A higher content of dietary fibers in AP, CP, and OP by-products could influence the increase in a^∗^ values of bread crumb. In addition, dietary fibers bound water, which is a medium for browning reactions between monosaccharides and amino acids (Kurek et al., 2017). Gil (2015) also found that coconut flour provided a reddish color to wheat/coconut bread crumb.

Acrylamide formation occurs through thermal reactions and involves reducing sugars and asparagine or aspartic acid, as well as the deamination and decarboxylation of asparagine (Nachi et al., 2018). The amounts of the previously mentioned compounds as well as the pH, water activity, and parameters of the heating process influence the occurrence of acrylamide in cooked or fried starchy foods. Changes in the acrylamide level in produced breads with AP, CP, and OP could be related to the reducing sugar and free asparagine content, as well as lipid oxidation (Kuek et al., 2020). Moreover, different treatments of additives may lead to different availabilities of reactions between acrylamide precursors. Oats may have higher acrylamide-forming potential than wheat due to increased levels of simple sugars and free asparagine (Žilić et al., 2017). In this study, the lowest acrylamide content was found in bread with OP probably due to that, the major part of these compounds was lost during plant drink preparation or consumed by yeast and LAB fermentation. The unsaturated fatty acids constitute about 90% of the total fatty acid content in almonds (Roncero et al., 2016). Therefore, this could be one of the reasons for the greater formation of acrylamide in bread with AP. The effect of ultrasonication on acrylamide production could be related to the increased solubility of proteins (Shen et al., 2017). It has been reported that the Maillard reaction could be impeded and acrylamide formation could be prevented due to decreased pH during fermentation (Nachi et al., 2018). On the other hand, the produced reducing sugars and other precursors of brown pigments, as well as the increased proteolysis through the fermentation process of plant drink preparation by-products, could intensify acrylamide formation in bread (Hayta and Ertop, 2019).

## Conclusion

This study showed that ultrasonication at 37 kHz and fermentation are both effective for improving the biosafety of by-products. The tested by-products had a different influence on the dough and bread quality parameters; however, the specific volume and porosity of the breads showed a very weak correlation with overall acceptability (*r* = 0.1210 and *r* = 0.1413, respectively), and the most acceptable breads were prepared with 20% fermented AP, 15% fermented OP, and 15% ultrasonicated OP. Also, the tested by-products influenced the bread staling process, and the lowest hardness (lower than that control bread) after 96 h of storage of the breads prepared with 5% fermented AP, CP, and OP (1.17, 1.13, and 1.30 mJ, respectively) and with 5% ultrasonicated CP (1.2 mJ) was found. The addition of the selected by-products to the main wheat bread formula could reduce acrylamide formation; a significantly lower acrylamide content was found in breads prepared with 5% and 10% ultrasonicated AP, compared with the control breads. However, the lowest acrylamide content in bread prepared with OP was found (except for breads prepared with 15% and 20% ultrasonicated OP). Finally, 15% fermented OP can be safely used for wheat bread preparation because the prepared bread showed high OA, as well as lower acrylamide concentrations than the control bread.

## Data Availability Statement

The original contributions presented in the study are included in the article/supplementary material, further inquiries can be directed to the corresponding author.

## Author Contributions

EB planned the experiment, performed the statistical analyses, and wrote the original draft. VB, IP, AB, EZ, VL, VS, and PZ carried out the experiment and performed all analyses. DK wrote the original draft and revised the manuscript. DZ and GJ revised the manuscript. All authors contributed to the article and approved the submitted version.

## Conflict of Interest

The authors declare that the research was conducted in the absence of any commercial or financial relationships that could be construed as a potential conflict of interest.
